# Lasing Emission from Soft Photonic Crystals for Pressure and Position Sensing

**DOI:** 10.3390/nano13222956

**Published:** 2023-11-15

**Authors:** Tsan-Wen Lu, Zhen-Yu Wang, Kuang-Ming Lin, Po-Tsung Lee

**Affiliations:** Department of Photonics, College of Electrical and Computer Engineering, National Yang Ming Chiao Tung University, Hsinchu 300093, Taiwan; stroowang@gmail.com (Z.-Y.W.); summer210576@gmail.com (K.-M.L.); potsung@nycu.edu.tw (P.-T.L.)

**Keywords:** photonic crystal, optical strain sensors, nanocavity, semiconductor lasers, flexible nanophotonics

## Abstract

In this report, we introduce a 1D photonic crystal (PhC) nanocavity with waveguide-like strain amplifiers within a soft polydimethylsiloxane substrate, presenting it as a potential candidate for highly sensitive pressure and position optical sensors. Due to its substantial optical wavelength response to uniform pressure, laser emission from this nanocavity enables the detection of a minimum applied uniform pressure of 1.6‰ in experiments. Based on this feature, we further studied and elucidated the distinct behaviors in wavelength shifts when applying localized pressure at various positions relative to the PhC nanocavity. In experiments, by mapping wavelength shifts of the PhC nanolaser under localized pressure applied using a micro-tip at different positions, we demonstrate the nanocavity’s capability to detect minute position differences, with position-dependent minimum resolutions ranging from tens to hundreds of micrometers. Furthermore, we also propose and validate the feasibility of employing the strain amplifier as an effective waveguide for extracting the sensing signal from the nanocavity. This approach achieves a 64% unidirectional coupling efficiency for leading out the sensing signal to a specific strain amplifier. We believe these findings pave the way for creating a highly sensitive position-sensing module that can accurately identify localized pressure in a planar space.

## 1. Introduction

Embedding various photonic meta-structures in deformable soft materials [1,2,3] has been a well-known architecture for realizing various functional devices in flexible photonics. By applying different stresses to the soft carrier materials, one can easily deform the entire structures, thereby altering the optical modes typically tailored using photonic crystals (PhCs) or metallic nanostructures with plasmonic resonance. This alteration allows for the creation of various tunable photonic devices [1,2,3] or optical sensors [3,4,5,6]. These changes in optical modes often manifest as shifts in optical wavelengths, which, in turn, result in modifications to other optical properties, including focal length [7,8], polarization, optical phase [9], color [10,11], intensity [12,13], etc. This facilitates the realization of a wide range of tunable functional devices suitable for various applications [14,15,16,17,18]. Concurrently, such alterations in optical wavelengths, presented as optical spectra [19,20,21,22] or visible color changes [23,24,25,26,27,28,29,30,31,32,33,34,35], are commonly employed for sensing or quantifying different types of stresses applied to this architecture. In recent years, there have been numerous reports on PhCs or metallic nanostructures with plasmonic resonance embedded in soft materials, designed for sensing various strain-related parameters such as physiological parameters [13,26,27], motions [28,29], uniform pressure [30,31,32], and different types of stress [20,21,22,33,34,35]. Most of these reports have demonstrated sensing via colorimetric changes in broad-band optical modes within large-area meta-structures [23,24,25,26,27,28,29,30,31,32,33,34,35]. In comparison, only a few have explored sensing via spectral variations in narrow-band optical modes within localized meta-structures [19,20,21,22]. The former approach offers the advantage of easily interpretable sensing results, visible to the naked eye, while the latter provides higher sensitivity for detecting minute variations in strain. Furthermore, it is worth noting that the majority of previously reported strain sensors have focused on uniformly applied stress. To the best of our knowledge, the sensing of locally applied stress in a planar space using this type of sensor has not been reported or thoroughly investigated up to this point.

Therefore, to address this gap in the research, we utilized our previously proposed 1D PhC nanocavity with strain amplifiers [36] and embedded it in a soft substrate made of polydimethylsiloxane (PDMS). This architectural configuration exhibits a significant optical wavelength response to uniform stress. Capitalizing on this characteristic, in this report, we explored its distinct wavelength shift behaviors when applying localized pressure at various positions relative to the PhC nanocavity. In addition to validating its potential to function as an optical position or touch sensor with micro-scale spatial resolution in experiments, we also conducted theoretical studies to explore the feasibility of using the strain amplifier as an effective waveguide for extracting the sensing signal.

## 2. Design of PhC Nanocavity Sensitive to Deformation

**Figure 1:**
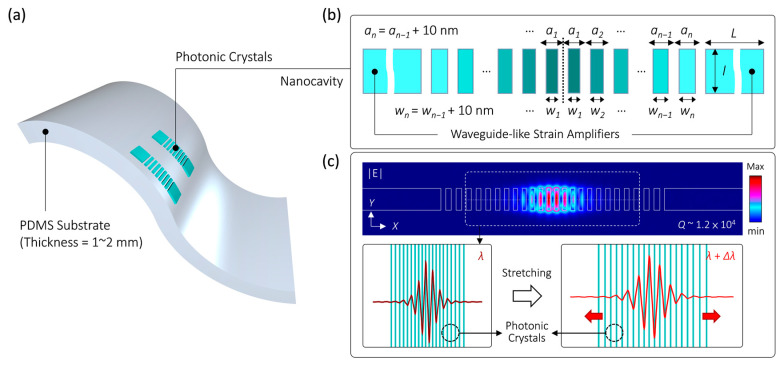
(**a**) Schematic of 1D PhCs in a PDMS substrate and (**b**) its nanocavity design with waveguide-like strain amplifiers. (**c**) Theoretical |E|-field distributions of the dielectric mode inside the nanocavity along the *XY* plane and a schematic of lattice expansion leading to mode stretching accompanied by a wavelength shift Δ*λ*.

Figure 1a depicts the schematic of 1D PhCs embedded in the deformable PDMS substrate investigated in this report. These PhCs comprise periodically spaced InGaAsP nanorods with a thickness of 240 nm along a single axis. Figure 1b provides additional details on the PhC lattice parameters, including a height (*H*) of 950 nm, a width (*w_n_*), and a lattice constant (*a_n_*). Both *w_n_* and *a_n_* gradually increase by 10 nm from the central region to both sides. This increase can be expressed as *w_n+_*_1_ = *w_n_* + 10 nm and *a_n+_*_1_ = *a_n_* + 10 nm, where *n*, *w*_1_, and *a*_1_ are 14, 135 nm, and 340 nm, respectively. This gradual lattice forms a double hetero-lattice structure that can serve as a nanocavity based on the mode-gap effect [37], enabling the local confinement of the photon flow propagating along the lattice direction. Additionally, waveguide-like strain amplifiers, previously reported by us [36], connect both ends of the PhCs. These amplifiers are employed to enhance the strain within the PhCs through the film edge-induced strain effect [38], increasing their optical response to the applied stress [36,39]. These strain amplifiers have a length denoted as *L* and are made of the same material as PhCs with the same height.

Figure 1c illustrates the theoretical electric field distributions along the *XY* plane of the dielectric mode well-confined within this nanocavity using the 3D finite-element method (FEM) in the COMSOL Multiphysics software package 3.5a. For InGaAsP and PDMS with refractive indices (*n_InGaAsP_* and *n_PDMS_*) of 3.4 and 1.4, respectively, the dielectric mode within the nanocavity exhibits a high-quality factor (*Q*) of 1.2 × 10^4^ and a small effective mode volume (*V_eff_*) of 0.76 (*λ*/*n_InGaAsP_*)^3^. These values result in a sufficiently high *Q*/*V_eff_* ratio of the dielectric mode, enabling strong light–matter interactions and the realization of a low-threshold nanolaser. Furthermore, as the PhCs are structurally discontinuous in this design, they can be easily deformed when various stresses are applied to the PDMS substrate. In particular, Figure 1c schematically demonstrates the alteration of the dielectric mode when stretching the PDMS substrate along the PhC lattice direction. In this scenario, the dielectric mode expands with the stretching of the PhCs, resulting in an increase in its optical wavelength. This wavelength increase can be used as a parameter to evaluate the magnitude of the applied stretching stress.

## 3. Manufacturing Process

**Figure 2:**
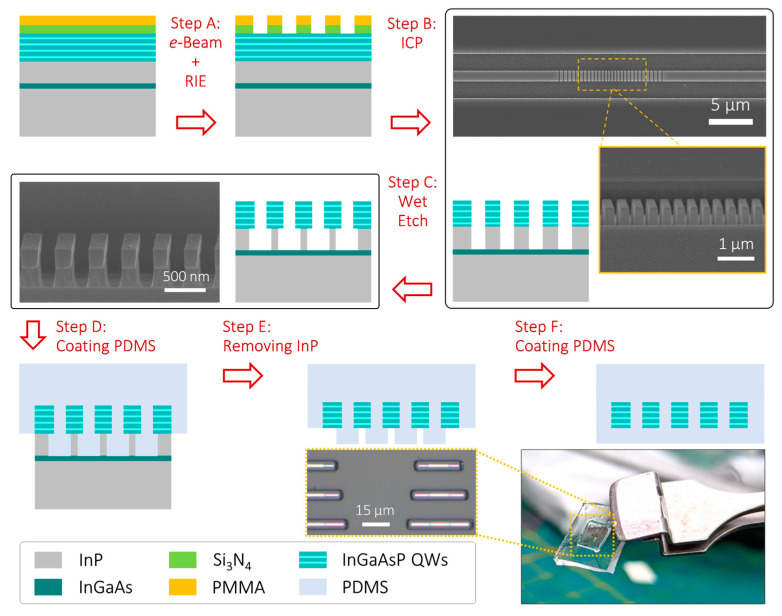
Flowchart of the manufacturing process for embedding PhC nanocavities with waveguide-like strain amplifiers in a PDMS substrate and SEM images or photographs of each step.

Figure 2 displays the flowchart of the manufacturing process for the design described above. The process initiates by defining the 1D PhC patterns on a SiN_x_ hard mask coated with an electron beam (*e*-beam) resisting polymethylmethacrylate (PMMA) on top of InGaAsP quantum wells (QWs) via *e*-beam lithography. Next, the patterns are transferred to the SiN_x_ hard mask using a reactive-ion etching (RIE) process (Step A). Subsequently, the patterns are further transferred to the InGaAsP QWs and the underlying InP substrate using an inductively coupled plasma (ICP) dry etching process (Step B). Top- and tilted-view scanning electron microscopy (SEM) images after Step B reveal the PhC lattices successfully manufactured on the InP substrate. The process then proceeds with partially removing the exposed underlying InP substrate by immersing the PhC lattices in a diluted HCl solution (HCl:H_2_O = 1:5) for 90 s at room temperature (Step C). This particular step results in the partially etched underlying InP substrate becoming fine posts that support the PhC lattices, as observed in the tilted-view SEM image after Step C. These InP posts serve the crucial purpose of ensuring the tight wrapping of the subsequent spin-coating of PDMS around the PhC lattices (Step D). For this step, we used Dow Sylgard^TM^ 184, with a mixing volume ratio of SylgardA to SylgardB of 10:1. After a 17 h bake at 60 °C, the underlying InP substrate is removed by immersing it in a diluted HCl solution (HCl:H_2_O = 3:1) for 1 h at room temperature (Step E). The optical microscope image shows nanocavities with various lengths of strain amplifiers inside the PDMS substrate following this step. Finally, a PDMS layer is spin-coated onto the exposed surface of the PhCs and baked to complete the entire process (Step F). Figure 2 displays a photograph of the PDMS substrate with nanocavities after the successful completion of Step F.

## 4. Measurement Results and Discussions

### 4.1. Laser Emission from PhC Nanocavity and Its Wavelength Response to Uniform Pressure

**Figure 3:**
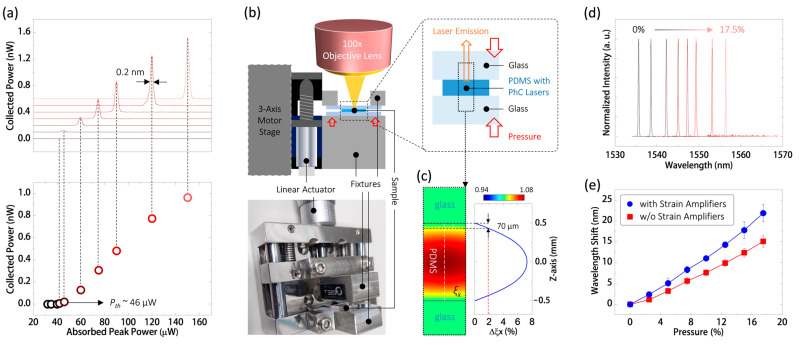
(**a**) (**Bottom**) Optical excitation curve of a PhC nanocavity embedded in a PDMS substrate, and (**Top**) corresponding spectra under different excitation powers. (**b**) Schematic and photograph of the homemade linear actuating stage for applying uniform pressure to the PDMS substrate clamped between two glass plates. (**c**) Theoretical *ξ_X_* distribution of the 1 mm thick PDMS substrate along the *XZ*-plane when subjected to a 10% uniform pressure applied along the *Z*-direction. (**d**) Lasing spectra of the nanocavity under varying uniform pressure from 0 to 17.5% were applied along the *Z*-direction. (**e**) Average lasing wavelength shifts of PhC nanocavities with and without waveguide-like strain amplifiers in a PDMS substrate under different applied uniform pressure.

In our measurements, we employed a 980 nm diode laser pulse with a pulse width of 20 ns and a 1.8% duty cycle to excite the PhC nanocavity shown in Figure 2 at room temperature. Figure 3a illustrates its optical excitation curve and the corresponding single-mode lasing spectra under various excitation peak powers ranging from 42 to 150 μW. The lasing threshold power (*P_th_*) of the nanocavity, estimated from the excitation curve, is approximately 46 μW. The spectral linewidth of the lasing emission, measuring about 0.2 nm, can offer sufficient spectral resolution for use as an optical sensor.

To observe the optical wavelength response of the PhC nanocavity to the applied uniform pressure in our measurements, we employed homemade fixtures and two glass slides to securely hold the PDMS substrate on a linear actuating stage, as illustrated in the configuration and image in Figure 3b. The linear actuator was used to apply uniform pressure to the sandwiched PDMS substrate by pushing the fixture. To understand the actual strain distribution within the 1 mm thick PDMS substrate in this setup, we calculated the strain distribution along the *X* direction (*ξ_X_*) using 3D FEM, as depicted in Figure 3c. In Figure 3c, *ξ_X_* exhibits a parabolic decrease from the center of the PDMS substrate to its top and bottom surfaces. When we applied a 10% pressure along the *Z*-direction, *ξ_X_* showed a 1.9% increment at a position 70 μm away from the top surface, where the PhC nanolaser is located. This increase in *ξ_X_* will lead to the stretching of the PhC lattices, as previously illustrated in Figure 1c. For the PhC nanocavity defined in Figure 1b, its theoretical optical wavelength response (*R_S_*) to each one percent *ξ_X_* increment is 10.6 nm. This means that a 10% pressure applied along the *Z*-direction results in a 20.1 nm wavelength shift of the dielectric mode inside. In other words, the theoretical optical wavelength response (*R_P_*) to each one percent uniform pressure is approximately 2.0 nm. Accordingly, the theoretical minimum detectable uniform pressure (Δ*P_det_*) can be evaluated using the following equation:

Δ*P_det_* = 1/*R_P_* × *λ*/*Q* (1)

Here, *λ*/*Q* represents the spectral linewidth of the dielectric mode in the nanocavity. For a theoretical *R_P_* of 2.0 nm/% and *λ*/*Q* of 0.128 nm, we can calculate the theoretical Δ*P_det_* to be 0.64‰ when used as an optical pressure sensor.

In the measurement, using the setup depicted in Figure 3b, Figure 3d displays typical lasing spectra of the PhC nanolaser in the PDMS substrate under uniformly applied pressure ranging from 0 to 17.5% along the *Z*-direction. This measurement is performed three times, and the average lasing wavelength shifts are recorded in Figure 3e. As predicted, the nanolaser exhibits a wavelength redshift caused by the lattice expansion due to the applied pressure. This wavelength shift gives an *R_P_* value of 1.25 nm/% via linear fitting. According to Equation (1), under a measured lasing spectral linewidth of 0.2 nm, the experimental Δ*P_det_* is approximately 1.6‰. The difference between theoretical and experimental Δ*P_det_* mainly arises from the discrepancy between the theoretical linewidth *λ*/*Q* and the experimental lasing spectral linewidth with thermal broadening. When further individually evaluating the sensitivity in each round of measurement, the *R_P_* values are 1.24, 1.35, and 1.22 nm/% via linear fitting in each round of measurement, which corresponds to experimental Δ*P_det_* values of 1.61, 1.48, and 1.64‰, respectively. This means there is a sensitivity uncertainty of 0.16‰, which is caused by the actuating stability limit of our mechanical linear actuating stage used to apply pressure in the measurement. For comparison, we also conducted the same measurement on the PhC nanolaser without strain amplifiers. In Figure 3e, it is evident that the *R_P_* value is significantly smaller, evaluated as 0.86 nm/% via linear fitting, compared to the results mentioned above. This comparison indicates the effectiveness of the strain enhancement provided by the waveguide-like strain amplifiers.

### 4.2. Wavelength Response to Localized Pressure for Position Sensing

**Figure 4:**
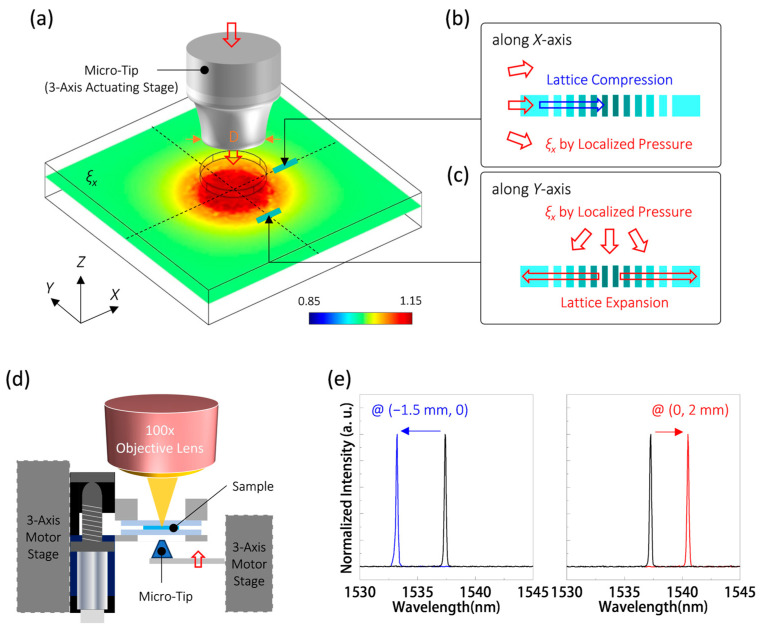
(**a**) Theoretical distribution of *ξ_X_* along the *XY* plane when localized pressure is applied to a 1 mm PDMS substrate using a micro-tip with *D* of 500 μm. Schematics of lattice deformations when localized pressure is applied near the PhC nanocavity along the (**b**) *X* and (**c**) *Y* directions. (**d**) Schematic of the measurement setup for applying localized pressure to the PDMS substrate using a 3-axis actuating-stage-controlled micro-tip. (**e**) Lasing spectra before and after applying localized pressure at the position coordinates of (−1.5 mm, 0) and (0, 2 mm) relative to the PhC nanocavity.

So far, we have demonstrated the significant optical wavelength response of the PhC nanolaser in the PDMS substrate proposed herein to uniform pressure. In this section, we further investigate the optical response when pressure is locally applied near the PhC nanolaser. Initially, Figure 4a illustrates the theoretical *ξ_X_* distribution of the PDMS substrate along the *XY* plane under localized pressure applied using a micro-tip with a tip diameter (*D*) of 500 μm. The distribution exhibits reasonably uniform *ξ_X_* within the region that overlaps with the tip and gradually weakens away from the tip region. When the PhC nanolaser is positioned at the center of the pressure-applied region by the tip, it experiences uniform pressure and exhibits a wavelength redshift, similar to the case demonstrated in Figure 3d. However, the situation changes for the PhC nanolaser located outside this region.

For the nanolasers situated at coordinates (1.5 mm, 0) and (0, −2 mm) away from the center of the tip region, as indicated in Figure 3a, the effects of localized pressure on PhC nanolasers will differ, as depicted in Figure 4b,c. Specifically, in Figure 4b, the *ξ_X_* caused by the localized pressure will compress the lattice of the PhC nanolaser located at (1.5 mm, 0), resulting in a wavelength blue shift of the dielectric mode. Conversely, in Figure 4c, the *ξ_X_* distribution expands the lattice of the PhC nanolaser located at (0, −2 mm), leading to a wavelength redshift of the dielectric mode.

In the measurement setup depicted in Figure 4d, a micro-tip with *D* of 500 μm, made of thermoplastic polyurethane, is affixed to a fixture and controlled using a 3-axis actuating stage. This micro-tip is then employed to apply localized pressure to different positions on the PDMS substrate from the back-side glass slide. Figure 4e displays the lasing spectra both before and after applying localized pressure at positions with coordinates (−1.5 mm, 0) and (0, 2 mm) relative to the PhC nanocavity. The PhC nanolaser exhibits a wavelength blue shift of 4.2 nm and a redshift of 3.3 nm, respectively, which is consistent with the responses predicted in Figure 4b,c.

**Figure 5:**
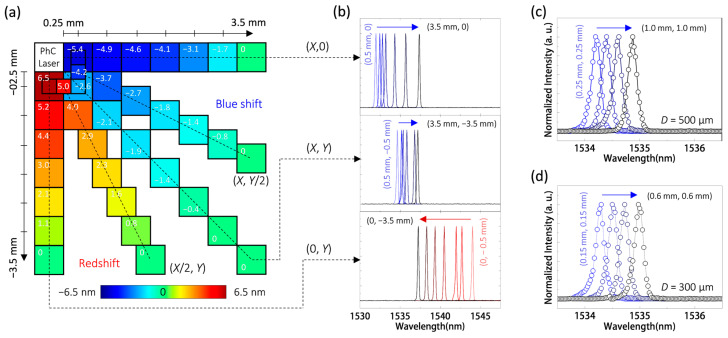
(**a**) Spatial distribution of wavelength shifts observed from the PhC nanolaser under localized pressure applied at different positions within quadrant *IV* (with a size of 3.5 mm × 3.5 mm) of the PhC nanolaser. (**b**) Lasing spectra of the PhC nanolaser as the micro-tip pressing moves from coordinates: (**Top**) (0.5 mm, 0) to (3.5 mm, 0), (**Middle**) (0.5 mm, −0.5 mm) to (3.5 mm, −3.5 mm), and (**Bottom**) (0, −0.5 mm) to (0, −3.5 mm). Lasing spectra of the PhC nanolaser when localized pressure is applied using micro-tips with (**c**) a *D* of 500 μm, moving from positions with coordinates (0.25 mm, −0.25 mm) to (1.0 mm, −1.0 mm), or with (**d**) a *D* of 300 μm, moving from positions with coordinates (0.15 mm, −0.15 mm) to (0.6 mm, −0.6 mm).

The results mentioned above indicate the feasibility of identifying localized pressure at various positions near the PhC nanolaser. To further validate this capability, we utilized the micro-tip to apply a 10% localized pressure at different positions within quadrant *IV* (with a size of 3.5 mm × 3.5 mm) of the PhC nanolaser. In Figure 5a, we present the spatial distribution of wavelength shifts observed from the PhC nanolaser due to the aforementioned pressure applied within quadrant *IV*. At first, when the micro-tip locally pressed the position with coordinates (0.5 mm, 0) relative to the PhC nanolaser, the nanolaser exhibited a wavelength blue shift of 5.4 nm. This wavelength shift decreased progressively as the micro-tip was moved away (with an increment of 0.5 mm) from the nanolaser along the *X* direction.

When the micro-tip was positioned at coordinates (3.5 mm, 0) and locally pressed, the PhC nanolaser displayed no wavelength shift anymore. On the other hand, a similar decrease in wavelength redshift, ranging from 6.5 to 0 nm, was also observed as the micro-tip pressing moved from positions with coordinates (0, −0.5 mm) to (0, −3.5 mm). Figure 5b depicts the corresponding lasing spectra of the PhC nanolaser during the micro-tip pressing motion described above.

In addition to movements along the *X* and *Y* directions, Figure 5a also presents results when the micro-tip pressing occurs within the region between the *X* and *Y* directions. When the micro-tip pressing moves along the direction with coordinates (*X*, *Y*) (where *X* = *Y* = 0.5–3.5 mm) away from the nanolaser, the nanolaser exhibits a gradually decreasing wavelength blue shift. However, from the lasing spectra shown in Figure 5b, it is evident that the magnitude of the wavelength blue shifts in this direction is significantly smaller than those observed along the *X* direction. In particular, the nanolaser demonstrates a wavelength blue shift of only 2.1 nm when pressed at the position with coordinates (0.5 mm, −0.5 mm). This phenomenon occurs because the wavelength shift along this direction is simultaneously influenced by both compression and stretching due to the localized pressure shown in Figure 4b,c. This inference can be further substantiated with the measurement results when the micro-tip pressing moves along a direction closer to either the *X* or *Y* direction. As illustrated in Figure 5a, when the micro-tip pressing moves along the direction with coordinates (*X*/2, *Y*) (where *X* = *Y* = 0.5–3.5 mm) away from the nanolaser, the nanolaser displays a wavelength redshift diminishing with distance. Along this direction, the localized pressure primarily causes lattice stretching of the nanolaser rather than compression. Conversely, for the scenario along the direction with coordinates (*X*, *Y*/2) (where *X* = *Y* = 0.5–3.5 mm) away from the nanolaser, the nanolaser exhibits more significant wavelength blue shifts than along the direction with coordinates (*X*, *Y*), and also decreases with distance. In this case, the localized pressure predominantly results in lattice compression of the nanolaser, as opposed to lattice stretching. Based on this position–position-dependent wavelength shift due to localized pressure, we believe it is feasible to employ a combination of the PhC nanolasers proposed here to create position sensors.

On the other hand, spatial resolution is one of the key properties of a position sensor. In Figure 5a,b, along the direction with coordinates (*X*, 0), the lasing spectra exhibit a wavelength redshift of 0.5 nm when the micro-tip pressing moves from the position with coordinate (0.5 mm, 0) to coordinate (1.0 mm, 0). This means the optical wavelength response to the micro-tip pressing position, denoted as *R_POS_*, is approximately 1.0 nm/mm. When considering the spectral resolution, *λ_res_*, provided by the lasing emission (which is 0.2 nm), we can calculate the minimum detectable micro-tip position difference (Δ*POS_det_*) to be 200 μm using a similar equation to Equation (1) expressed as

Δ*POS_det_* = 1/*R_POS_* × *λ_res_* (2)

However, when the micro-tip pressing moves from positions with coordinates (3.0 mm, 0) to (3.5 mm, 0), the 1.7 nm wavelength shift between these two positions produces a larger *R_POS_* value of 3.4 nm/mm. This *R_POS_* value results in a Δ*POS_det_* of 60 μm under *λ_res_* = 0.2 nm. Therefore, the minimum detectable position differences are position-dependent and range from 60 to 200 μm along the *X* direction. Similar position-dependent Δ*POS_det_* values of tens to hundreds of micrometers can also be observed when the micro-tip pressing moves along the directions with coordinates (0, *Y*), (*X*/2, *Y*), (*X*, *Y*), and (*X*, *Y*/2).

To further minimize the Δ*POS_det_* value, one straightforward approach is to increase the localized pressure from the current 10% to 15% or more. In this scenario, the wavelength shifts will be enhanced at all positions, further improving the spatial resolution. Additionally, the spatial resolution also depends on the size of the localized pressure. Figure 5c presents the lasing spectra of the PhC nanolaser when the micro-tip pressing moves along the direction with coordinates (*X*, *Y*), where *X* = *Y* = 0.25, 0.5, 0.75, and 1.0 mm. The total wavelength shift of 0.8 nm within a displacement of the micro-tip by 0.75√2 mm means an *R_POS_* value of 0.76 nm/mm, resulting in a Δ*POS_det_* value of 260 μm under *λ_res_* = 0.2 nm. By using a micro-tip with a *D* of 300 μm, Figure 5d displays the lasing spectra of the PhC nanolaser when the micro-tip pressing moves along the direction with coordinates (*X*, *Y*), where *X* = *Y* = 0.15, 0.3, 0.45, and 0.6 mm. The lasing spectra show a wavelength redshift of 0.8 nm within a shorter displacement of 0.45√2 mm, resulting in a larger *R_POS_* of 1.26 nm/mm, which refines the Δ*POS_det_* to be 160 μm under *λ_res_* of 0.2 nm.

### 4.3. Waveguide-like Strain Amplifier for Leading out the Sensing Signal

**Figure 6:**
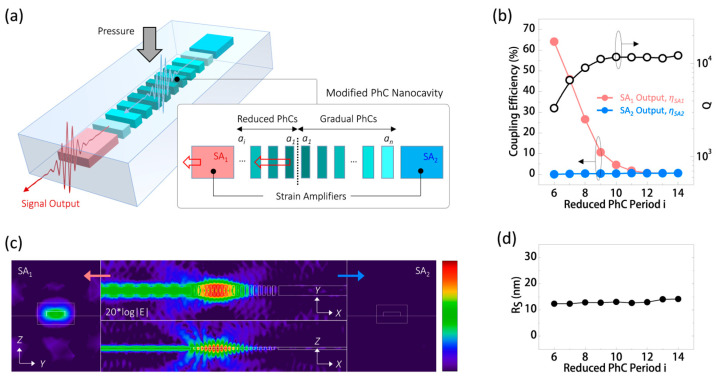
(**a**) Schematic of a PhC nanocavity with a reduced PhC lattice period on one side for unidirectional coupling energy within the nanocavity to a specific waveguide-like strain amplifier, SA_1_. (**b**) Theoretical *Q*, *η_SA_*_1_, and *η_SA_*_2_ of the dielectric mode in the PhC nanocavity with different *i* ranging from 14 to 6. (**c**) For *i* = 6, the theoretical logarithmic-scale |E|-field distributions (along the *XY* and *XZ* planes) along the nanocavity, as well as the electric fields at the outputs of strain amplifiers SA_1_ and SA_2_ (along the *YZ* plane). (**d**) Theoretical *R_S_* of the dielectric mode under different *i* values ranging from 14 to 6.

As an optical sensing module, efficiently extracting the optical sensing signal is another crucial consideration. In our proposed design, apart from enhancing strain within the PhC nanocavity region, the strain amplifier can also serve as a waveguide to lead out the energy of the dielectric mode within the nanocavity. To initially validate this possibility, we made slight modifications to the PhC nanocavity design. In Figure 6a, we reduced the PhC lattice period (*i*) from 14 to 6 on one side (SA_1_) to weaken its photonic band gap confinement, allowing mode energy to leak into the waveguide-like strain amplifier on this side. At various reduced PhC periods, Figure 6b presents the variations in the theoretical *Q* factor of the modified nanocavity and the variation in the coupling efficiencies (*η_SA_*_1_ and *η_SA_*_2_) to waveguide-like strain amplifiers SA_1_ and SA_2_. Here, *η_SA_*_1_ and *η_SA_*_2_ represent the energy coupling ratio between the respective strain amplifier outputs and all the boundaries in the simulation domain. In Figure 6b, when *i* is greater than or equal to 10, the *Q* value remains nearly constant at around 1.2 × 10^4^. This high *Q* value indicates poor energy leakage to the strain amplifiers SA_1_ and SA_2_, resulting in low *η_SA_*_1_ and *η_SA_*_2_ values, both below 5%. However, as *i* decreases below 10, *η_SA_*_1_ increases significantly with the rapid decrease in *Q*. For *i* = 6, a theoretical *η_SA_*_1_ value of 64% can be achieved while maintaining a sufficiently high *Q* of 3300 for low-threshold lasing. Meanwhile, the *η_SA_*_2_ value remains lower than 0.1%, ensuring highly unidirectional coupling of the sensing signal and preventing unnecessary energy loss from the nanocavity. This unidirectional coupling is clearly illustrated by the theoretical logarithmic-scale |E|-field distributions along the nanocavity (along the *XY* and *XZ* planes) and at the strain amplifier outputs SA_1_ and SA_2_ (along the *YZ* plane), as shown in Figure 6c. Furthermore, Figure 6d presents the theoretical *R_S_* value of the PhC nanocavity under different *i*. The *R_S_* remains almost constant for cases with reduced lattice periods, ensuring its performance in sensing applied pressure with this modified PhC nanocavity.

## 5. Conclusions

In this study, we explored the optical properties of a 1D PhC nanocavity with waveguide-like strain amplifiers within a soft PDMS substrate. Initially, we investigated its optical properties under uniform pressure both in simulations and experiments, revealing a significant optical wavelength response. This response allows for the detection of a minimum uniform pressure of 1.0‰ in simulations and 1.6‰ in experiments when used as a pressure sensor. Building upon this feature, we further examined the effects of applying localized pressure near the PhC nanocavity. We used mechanical simulation results to explain the different behaviors in wavelength shifts when applying localized pressure at various positions relative to the PhC nanocavity. In experiments, we demonstrated a spatial mapping of wavelength shifts in the PhC nanolaser under localized pressure applied using a micro-tip at different positions, which matched with simulation predictions. As a highly sensitive position sensor, this spatial wavelength shift mapping enables the detection of position differences as small as tens to hundreds of micrometers, a metric that can be further improved by enhancing and minimizing the localized pressure. Additionally, we also proposed the possibility of utilizing the strain amplifier as the waveguide for extracting the sensing signal from the nanocavity, a crucial aspect in constructing it as an optical sensing module. By reducing one-side lattice periods of the PhC nanocavity, we achieved a 64% unidirectional coupling efficiency for extracting the sensing signal to a specific strain amplifier, all while maintaining the sensing properties for pressure measurements. Based on these findings, we believe it is feasible and potentially promising to utilize a combination of the PhC nanolasers proposed in this study to create a highly sensitive position-sensing module capable of accurately identifying localized pressure in planar space.

## Data Availability

Data are contained within the article.
